# Multiple Retinal Axons Converge onto Relay Cells in the Adult Mouse Thalamus

**DOI:** 10.1016/j.celrep.2015.08.003

**Published:** 2015-08-28

**Authors:** Sarah Hammer, Aboozar Monavarfeshani, Tyler Lemon, Jianmin Su, Michael Andrew Fox

**Affiliations:** 1 Virginia Tech Carilion Research Institute, Roanoke, VA 24016, USA; 2 Department of Biological Sciences, Virginia Tech, Blacksburg, VA 24061, USA; 3 Department of Pediatrics, Virginia Tech Carilion School of Medicine, Roanoke, VA 24016, USA; 4 Faculty of Health Sciences, Virginia Tech, Blacksburg, VA 24061, USA

## Abstract

Activity-dependent refinement of neural circuits is a fundamental principle of neural development. This process has been well studied at retinogeniculate synapses—synapses that form between retinal ganglion cells (RGCs) and relay cells within the dorsal lateral geniculate nucleus. Physiological studies suggest that shortly after birth, inputs from ~20 RGCs converge onto relay cells. Subsequently, all but just one to two of these inputs are eliminated. Despite widespread acceptance, this notion is at odds with ultrastructural studies showing numerous retinal terminals clustering onto relay cell dendrites in the adult. Here, we explored this discrepancy using brainbow AAVs and serial block face scanning electron microscopy (SBFSEM). Results with both approaches demonstrate that terminals from numerous RGCs cluster onto relay cell dendrites, challenging the notion that only one to two RGCs innervate each relay cell. These findings force us to re-evaluate our understanding of subcortical visual circuitry.

## Graphical Abstract

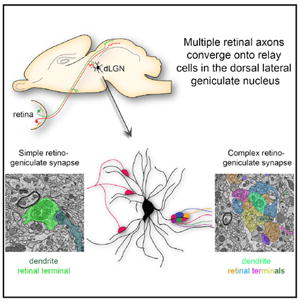

## INTRODUCTION

Initially, an exuberant number of axons generate synapses with target neurons in the brain only to have a large number of these supernumerary inputs eliminated in an activity-dependent fashion. This process, termed synapse elimination, has been well studied in the mouse visual thalamus, where synapses form between retinal projection neurons (i.e., retinal ganglion cells [RGCs]) and thalamic relay cells within the dorsal lateral geniculate nucleus (dLGN). Physiological studies over the past decade have suggested that retinal inputs to dLGN relay cells undergo an extensive amount of refinement during early postnatal development. While as many as 20 RGCs may innervate relay cells in the first week of mouse development, this number is reduced to just one to two RGCs by the end of the third postnatal week of rodent development (Chen and Regehr 2000; Jaubert-Miazza et al., 2005; Hooks and Chen 2006; Hong and Chen 2011) Furthermore, single-electrode recordings in mature primate LGN have been interpreted to indicate that retinal-derived excitatory postsynaptic potentials in relay cells arise from just one RGC (Sincich et al., 2007). Based upon these studies and the near unitary matching of retinal input to thalamic relay cell in the adult dLGN, the retinogeniculate synapse has emerged as a model for our understanding of activity-dependent refinement in the brain.

It is important to note, however, that these conclusions appear at odds with a series of 4-decade-old ultrastructural studies that characterized two distinct types of retinogeniculate synapses in dLGN: “simple encapsulated” retinogeniculate synapses are composed of single, large retinal terminals that contact large diameter relay cell dendrites and “complex encapsulated” retinogeniculate synapses that are composed of as many as ten distinct retinal terminals all converging on the same region of relay cell dendrite (Jones and Powell 1969; Guillery and Scott 1971; Lund and Cunningham 1972). If multiple retinal terminals converge at these synaptic sites, how can a near unitary matching of RGC axons to relay cells exist? A recent study by Hong et al. began to shed light on this paradox by revealing that the dramatic decrease in retinal convergence onto relay cells was accompanied by retinal terminals from single axonal arbors clustering onto postsynaptic sites in dLGN (Hong et al., 2014). This suggests that the numerous retinal terminals in “complex encapsulated” retinogeniculate synapses arise from branches of the same terminal arbor. To test this hypothesis, we employed “brainbow” adeno-associated viral vectors (brainbow AAVs) (Cai et al., 2013), a technique that permits the differential labeling of RGCs and their terminals with unique combinations of fluorescent reporter proteins. To our surprise, this approach revealed that clusters of retinal terminals originated from numerous, distinct RGCs. To test whether these clustered retinal terminals represented true synaptic connections with the same relay cell, we used serial block face scanning electron microscopy (SBFSEM), a technique that permits the 3D reconstruction of pre- and postsynaptic elements at high resolution (Denk and Horstmann, 2004). These analyses provide further evidence that terminals from numerous axons converged onto the same dendrite in “complex encapsulated” retinogeniculate synapses. These results challenge the notion that only one to two RGCs contact each dLGN relay cell and suggest we need to re-evaluate our understanding of the anatomy and development of subcortical visual circuitry.

## RESULTS

**Figure 1:**
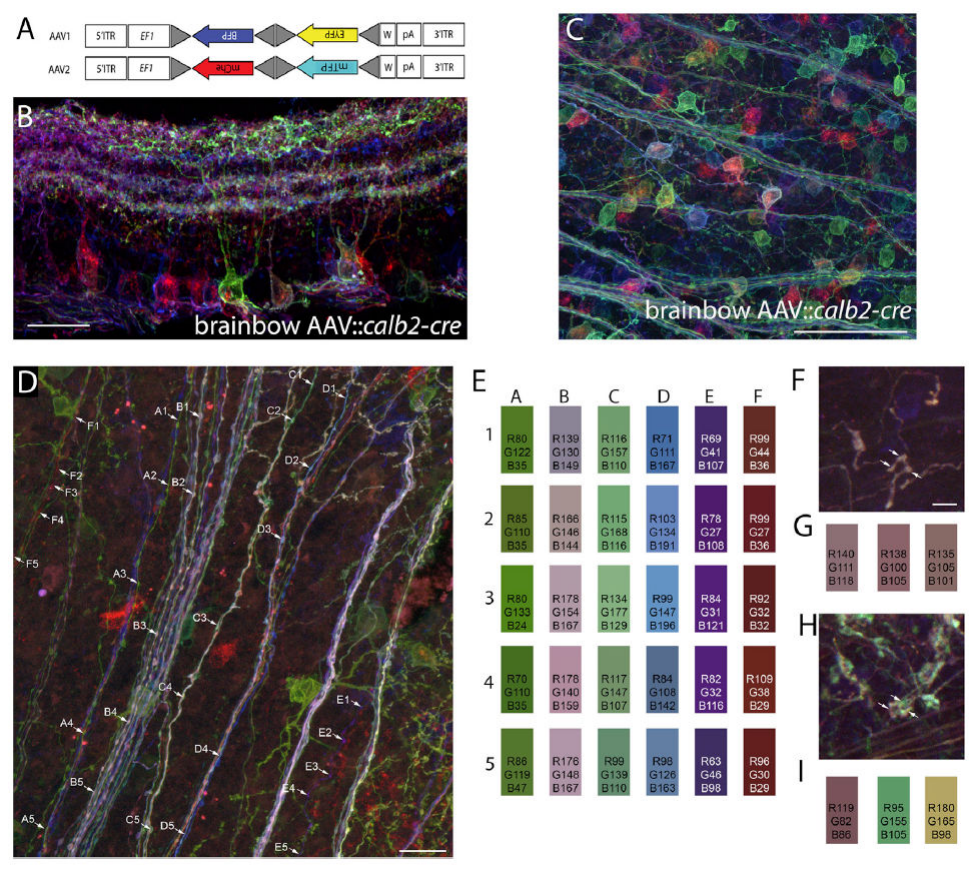
Labeling of RGCs and Retinal Axons with Brainbow AAVs (A) Schematic representing the constructs of each of the two brainbow AAVs used in these studies. Following Cre recombination, these two constructs generate either farnesylated Tag-blue fluorescent protein (BFP) or enhanced yellow fluorescent protein (EYFP), or monomeric Cherry fluorescent protein (mChe) or monomeric teal fluorescent protein (mTFP). EF1 represent regulatory elements from the elongation 1α gene and W represents elements from the woodchuck hepatitis virus posttranscriptional regulatory element. Lox site mutants are depicted with gray triangles. For additional details, see Cai et al. (2013). (B) Confocal image of a P35 retinal cross-section following intraocular injection of brainbow AAV into *calb2-cre* mice. Note the ability to delineate the dendritic arbor of the green-labeled RGC from adjacent fluorescently labeled RGCs. (C) Confocal image of a P35 retinal whole mount following intraocular injection of brainbow AAV into *calb2-cre* mice. (D) Confocal image of differentially labeled RGC axons in a P35 retinal whole-mount brainbow AAV∷*calb2-cre* mouse. (E) Color analysis at five locations (1–5) along the six axons labeled in (D) (labeled A–F). The color boxes represent the colors at each point highlighted along the axons. Numbers in the boxes represent the red (R), green (G), and blue (B) color intensity values at each point along the axons. Note the relative similar distribution of “color” along each axon. (F and G) A single retinal axon labeled with brainbow AAVs in the “core” region of dLGN of a P35 *calb2-cre* mouse. (G) Color analysis for the three boutons highlighted by arrows in (F). (H and I) Terminals from three distinct retinal axons converging at a single cluster following labeling with brainbow AAVs in the “core” region of dLGN of a P35 *calb2-cre* mouse. (I) Color analysis for the three boutons highlighted in (H). Scale bar in (B), 50 μm, in (D), 50 μm, in (C), 100 μm and in (F), 6 μm for (F) and (H).

**Figure 2:**
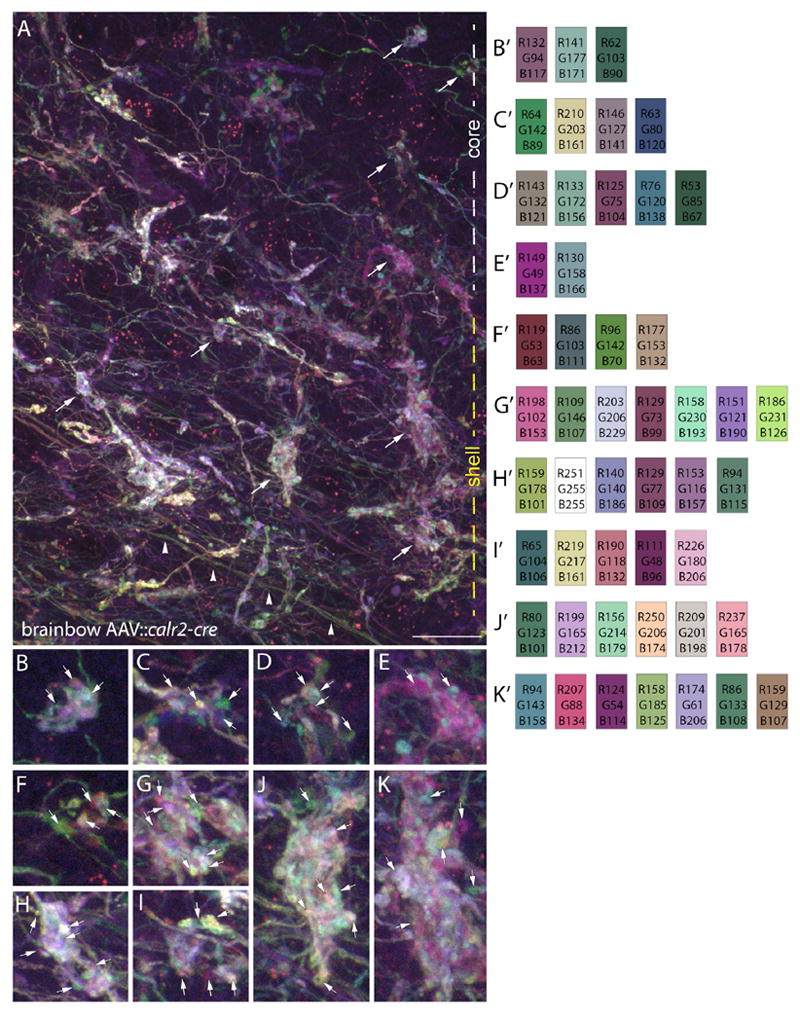
Clusters of Retinal Terminals in dLGN Contain Boutons from Multiple Retinal Axons (A) Maximum projection, confocal image of retinal axons, and terminals labeled with brainbow AAVs in the “core” and “shell” region of dLGN of P35 *calb2-cre* mice. White and yellow dashed lines on the right indicate the “core” and “shell” regions of dLGN in this image. Arrowheads highlight retinal axons traversing this region of dLGN. (B–K) High-magnification images of the retinal boutons indicated by arrows in (A). B′–K′ show color analysis for terminals highlighted with arrowheads in (B)–(K). Scale bar in (A), 20 μm for (A) and 7 μm for (B)–(K).

To assess whether clusters of retinal terminals originate from single RGC axons, we employed “brainbow” AAVs to differentially label RGCs with unique combinations of four fluorescent proteins—farnesylated Tag-blue fluorescent protein (BFP), enhanced yellow fluorescent protein (EYFP), monomeric Cherry fluorescent protein (mChe), and monomeric teal fluorescent protein (mTFP) (Cai et al., 2013). Brainbow-based technologies have previously been used successfully to trace axonal projections, including retinal projections, in a variety of vertebrate species (Robles et al., 2013; Livet et al., 2007; Pan et al., 2013; Cai et al., 2013). Each brainbow AAV is capable of driving the expression of two different fluorescent proteins (see Figure 1A); however, as each cell may express different levels of all four fluorescent proteins, a nearly limitless possibility of colors is achievable with these constructs (Cai et al., 2013). Since brainbow AAVs are Cre dependent, we injected a 1:1 mixture of both brainbow AAVs into the vitreous chamber of postnatal day 12–14 (P12–14) *calb2-cre* transgenic mice (Taniguchi et al., 2011). Cre recombinase is expressed by a large subset of RGCs in these mice (Zhu et al., 2014). After 3 weeks, mice were euthanized and RGCs were examined with confocal microscopy. Analysis in retinal cross-sections and whole mounts revealed three important points: (1) intraocular delivery of brainbow AAVs successfully labeled a large number of RGCs in *calb2-cre* mice (referred to here as brainbow AAV∷*calb2-cre* mice) (Figures 1B and 1C); (2) by examining dendritic stratification of uniquely labeled RGCs in brainbow AAV∷*calb2-cre* mice, it was clear that multiple classes of RGCs were labeled in these mice; (3) unique combinations of fluorescent reporter proteins were evenly distributed throughout retinal axons in brainbow AAV∷*calb2-cre* mice. This point is of particular importance since a key requirement for this “brainbow”-based approach to be successful is that there must be a uniform distribution of the entire constellation of fluorescent proteins within the axon and terminal arbor (so that color can be used as a marker of RGC origin of an axon). Previous studies on zebrafish RGCs have demonstrated that the distribution of brainbow-based fluorescent proteins remain uniformly distributed in both axonal and terminal compartments of the same cell (Robles et al., 2013). To be entirely sure that this was the case for retinal projections in mice, we analyzed color distribution in RGC axons in retinal whole mounts of brainbow AAV∷*calb2-cre* mice. Data presented here confirmed that the distribution of the entire constellation of fluorescent molecules expressed by a single RGC in brainbow AAV∷*calb2-cre* mice was evenly distributed throughout RGC axons in the retina (Figures 1D and 1E). These data support the feasibility of using differential labeling of RGC terminals in brainbow AAV∷*calb2-cre* dLGN to probe whether “complex encapsulated” retinogeniculate synapses arise from branches of the same terminal arbor.

To answer whether clusters of retinal terminals in dLGN arise from the same RGC, we turned our attention to the dLGN of P35 brainbow AAV∷*calb2-cre* mice. It is important to note that only a sparse population of retinal axons and terminals in dLGN were labeled with this approach (Figure 2). Regardless of this sparse labeling, we discovered that most terminal clusters contained numerous uniquely colored elements (Figures 1H, 1I, and 2). Since the “core” and “shell” regions of dLGN contain distinct types of relay cells and receive input from distinct types of RGCs (Dhande and Huberman 2014; Hong and Chen 2011; Krahe et al., 2011), we addressed whether multi-colored terminal clusters were present in both regions of P35 brainbow AAV∷*calb2-cre* mice dLGN. Analysis with anterograde tracers and immunohistochemistry with retinal-terminal specific markers both suggested that terminal clusters were present in both regions, but that clusters appeared larger and more widespread in the “shell” region of dLGN where axons from direction-selective RGCs arborize (Figure S1). Analysis in the P35 brainbow AAV∷*calb2-cre* mice revealed multi-colored terminal clusters in both “core” and “shell” regions of dLGN (Figures 1H and 2).

In contrast to the diversity of uniquely colored elements in these terminal clusters, retinal axons traversing this region of dLGN displayed consistent distributions of fluorescent proteins (see arrowheads in Figure 2A). This suggests that the numerous colored elements at terminal clusters represented axonal terminals originating from different RGCs. Importantly, when we analyzed individual retinal arbors in dLGN, we found uniform expression of fluorescent proteins in each bouton of a single retinal axon arbor (Figures 1F and 1G), again suggesting that terminal clusters containing multiple differently colored retinal terminals reflected clusters containing terminals from multiple RGCs.

While these results indicate that clusters of retinal terminals originate from multiple RGCs, they do not indicate whether these boutons contact the same dendrite or even the same relay cell. The gold standard for identifying such synaptic connections is with the use of high-resolution electron microscopy (EM)—the technique that first identified the complex encapsulated retinogeniculate synapses more than 4 decades ago (Jones and Powell 1969; Guillery and Scott 1971; Lund and Cunningham 1972). The problem with traditional EM approaches has been the difficulty in obtaining large volumes of serially sectioned EM images and in aligning and registering all of these images. Without large volumes of serially sectioned tissue, it has been impossible to identify the axonal origins of each presynaptic bouton at these complex retinogeniculate synapses. For this reason, we applied Serial Block Face Scanning Electron Microscopy (SBFSEM) to reconstruct retinogeniculate synapses at high resolution (5 nm/pixel) in P42 mouse dLGN. In electron micrographs, retinal terminals were identified based on the presence of pale-colored mitochondria and dense clusters of spherical synaptic vesicles; relay cell dendrites were identified based on the lack of synaptic vesicles, dark mitochondria, and microtubules; and synaptic sites between terminals and dendrites were identified by the presence of an identifiable active zone (Lund and Cunningham, 1972; Bickford et al., 2010; Hammer et al., 2014).

**Figure 3:**
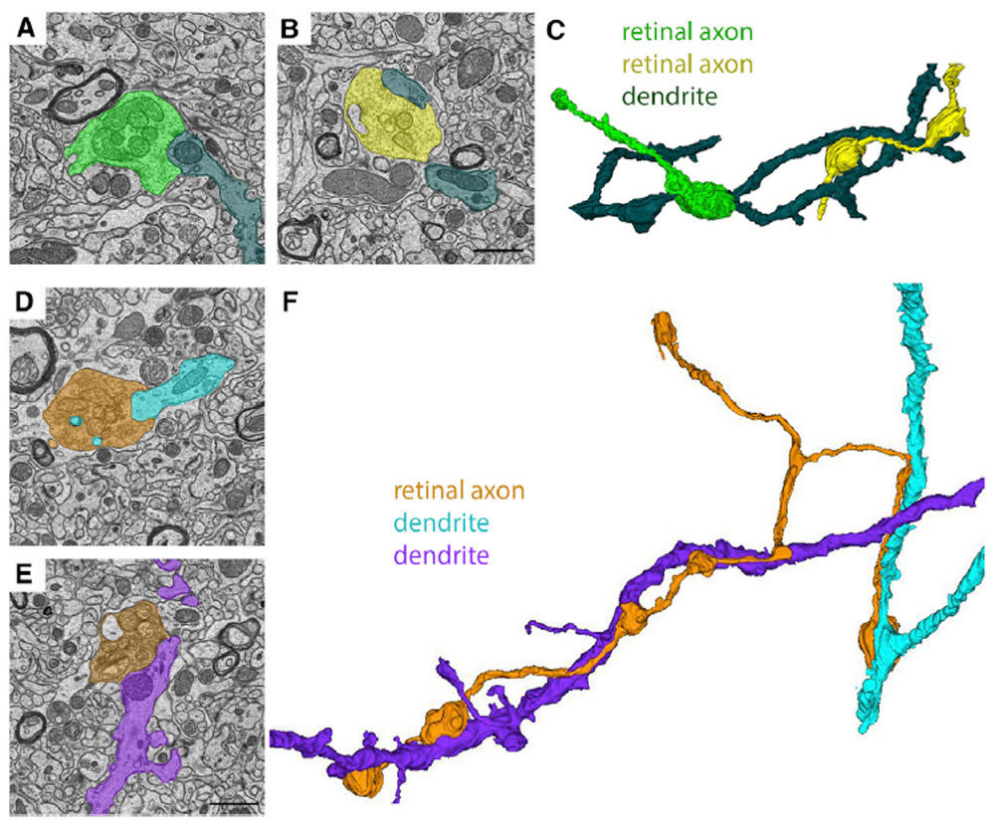
Ultrastructural Analysis and Reconstruction of Retinal Axons Contributing to “Simple Encapsulated” Retinogeniculate Synapses in dLGN (A and B) SBFSEM images of two retinal terminals synapsing onto the same relay cell dendrite in the “shell” region of dLGN. (C) 3D reconstruction of the two RGC terminal boutons from (A) and (B) converging on a single relay cell dendrite. (D and E) SBFSEM images of two retinal terminals from the same RGC axon making synaptic contact with two distinct relay cell dendrites in the “shell” region of dLGN. (F) 3D reconstruction of the retinal axon and relay cell dendrites from (D) and (E). Scale bar in (B), 1.5 μm for (A) and (B), and in (E), 1.5 μm for (D) and (E).

In total 71 retinal axons (and all of their terminal boutons within the volumes of tissue) were traced and reconstructed in the “shell” region of dLGN. These reconstructed axons generated 344 distinct synaptic sites onto the dendrites of relay cells and the average length of axon traced was 27.6 μm (±22 μm [SD]). Importantly, we observed single dendrites that received input from both simple and complex retinogeniculate synapses, suggesting single relay cells have the potential to be innervated by both types of retinal synapses (data not shown). Terminal boutons from 17 of these retinal axons participated in large, “simple encapsulated” retinogeniculate synapses (see Figure 3). Although only one of these axons generated terminal boutons that participated in both “simple encapsulated” retinogeniculate synapses and “complex encapsulated” retinogeniculate synapses (data not shown), this observation suggests that RGCs have the potential to generate both types of retinogeniculate synapses. Of the remaining 16 axons that generated large, “simple encapsulated” synapses, not a single example was identified in which terminals clustered around a single segment of dendrite. Instead, we observed single axons contacting multiple dendrites (Figures 3D–3F) and multiple retinal axons converging onto different regions of the same dendrite (Figures 3A–3C). The longest reconstructed retinal axon that contributed to simple retinogeniculate synapses measured 116.9 μm in length. It is important to point out, however, that in both cases it remains possible that these axons or dendrites may have originated from branches of the same RGC or relay cell, respectively, that branched outside of the volume of tissue reconstructed.

**Figure 4:**
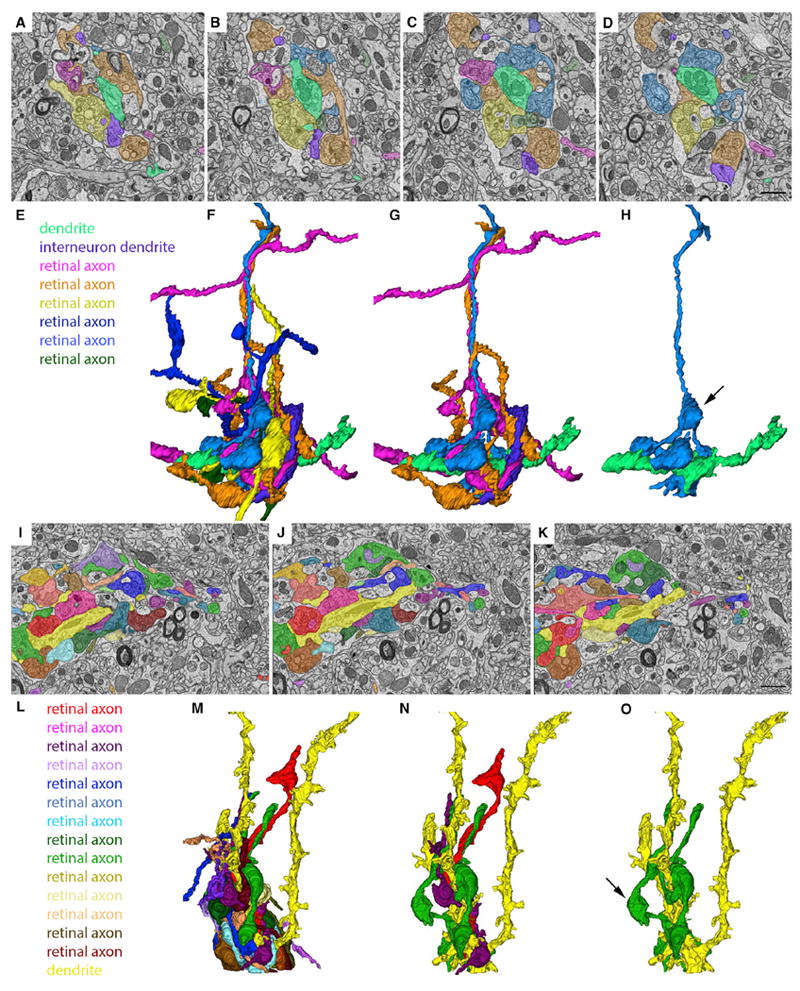
Ultrastructural Analysis and Reconstruction of Retinal Axons Contributing to “Complex Encapsulated” Retinogeniculate Synapses in dLGN (A–D) SBFSEM images of six retinal terminals synapsing onto the same relay cell dendrite (pseudo-colored in bright green) in the “shell” region of dLGN. (E) Key indicates the types of cellular elements pseudo-colored in (A)–(D) and (F)–(H). (F) 3D reconstruction of all of the elements pseudo-colored in (A)–(D). (G) 3D reconstruction of three RGC axons, an inhibitory interneuron dendrite and the relay cell dendrite in (A)–(D). (H) 3D reconstruction of a single RGC axon and the relay cell dendrite in (A)–(D). Arrow indicates a retinal bouton that makes synaptic contact with an element other than the relay cell dendrite pseudo-colored bright green. (I–K) SBFSEM images of 14 retinal terminals synapsing onto the same relay cell dendrite (pseudo-colored in bright yellow). (L) Key indicates the types of cellular elements pseudo-colored in (I)–(K) and (M)–(O). (M) 3D reconstruction of all of the elements pseudo-colored in (I)–(K). (N) 3D reconstruction of three RGC axons and the relay cell dendrite in (I)–(K). (O) 3D reconstruction of a single RGC axon and the relay cell dendrite in (I)–(K). Arrow indicates a retinal bouton that makes synaptic contact with an element other than the relay cell dendrite pseudo-colored bright yellow. Scale bar in (D), 1.5 μm for (A)–(D), and in (K), 1.5 μm for (I)–(K).

The remaining 54 retinal axons reconstructed generated boutons that contributed to “complex encapsulated” retinogeniculate synapses (Figure 4). In contrast to those retinal axons that generated “simple encapsulated” synapses, these 54 axons generated clusters of boutons that made synaptic contact with multiple, adjacent regions of the same dendrite (see blue and orange retinal terminals in Figures 4A–4H and bright green retinal terminals in Figures 4I–4O). The longest reconstructed retinal axon that contributed to complex retinogeniculate synapses measured 80.6 μm in length. These axons also generated boutons that made synaptic contact onto inhibitory interneuron dendrites (which were distinguished from relay cell dendrites by the presence of synaptic vesicles) (Lund and Cunningham 1972; Bickford et al., 2010) that were also identified in “complex encapsulated” synapses (see orange retinal terminals and purple inhibitory dendrite in Figures 4A–4H).

Although the bulk of SBFSEM analysis was performed on the “shell” region of dLGN, we also generated data sets from the “core” region of dLGN. Both simple and complex retinogeniculate synapses were observed in these data sets and boutons in the complex synapses appeared to originate from distinct retinal axons (Figure S1).

Next, we quantified different features of simple and complex retinogeniculate synapses. We found that retinal boutons participating in complex retinogeniculate synapses were smaller than their counterparts in simple synapses (Figure S2). Moreover, boutons in these complex synapses contained ~50% fewer active zones and ~50% fewer dendritic protrusions than boutons in simple retinogeniculate synapses (Figure S2). These differences were not merely the result of terminals being smaller in size, since there was a statistically significant difference in the active zone: bouton diameter ratio at these two synapse types (Figure S2).

While these results were expected based on previous studies (Lund and Cunningham, 1972; Bickford et al., 2010; Hong et al., 2014), the number of retinal axons that contribute to these clusters of retinal terminals was unexpected. In most cases, we observed terminal boutons originating from three to eight different retinal axons in these reconstructions (Figures 4A–4H); however, in a few cases we observed clusters that contained boutons from more than a dozen different retinal axons (Figures 4I–4O). While results from single retinal axon tracing studies could be interpreted to indicate that these clustered terminals originated from distant branches of one or two retinal axons (Hong et al., 2014; Dhande et al., 2011), our own studies of terminal clusters in brainbow AAV∷*calb2-cre* mice suggest that many of these terminal boutons do in fact originate from distinct RGCs (Figure 2). Therefore, taken together, these results and those described above in brainbow AAV∷*calb2-cre* mice indicate that numerous retinal inputs converge onto relay cells in the adult mouse dLGN.

## DISCUSSION

The retinogeniculate synapse has emerged as one of the most widely used models for studying activity-dependent refinement in the developing brain. In mice, physiological studies have suggested that as retinal axons initially innervate dLGN as many as 20 distinct RGC inputs converge onto the dendrites of relay cells, but, over the first few weeks of postnatal development, most supernumerary retinal inputs are eliminated so that each relay cell receives input from just one to two RGCs in the adult animal (Chen and Regehr 2000; Hong and Chen 2011). A concept so well entrenched, it has made its way into widely used textbooks (Squire et al., 2013).

Here, we applied two relatively novel technologies to examine the clustering of retinal terminals at retinogeniculate synapses. Our hope was to provide an explanation for why “complex encapsulated” retinogeniculate synapses exist if each relay cell receives input from just one to two RGCs. While our initial hypothesis was that each retinal terminal within a “complex encapsulated” retinogeniculate synapse originated from branches of a single RGCs, our data indicate that presynaptic terminals from numerous retinal axons converge onto relay cells in the mature rodent dLGN. These results raise an obvious question, what is the actual number of RGCs that innervate each relay cell? Unfortunately, an exact number cannot be determined since the studies described here focused on small regions of retinal arbors or dendritic trees, limiting the ability to reconstruct every single axon that synapses onto a given relay cell. That being said, for many relay cells the number of retinal inputs likely will approach or exceed a dozen. While this statement (and the results demonstrated here) contrasts the widely accepted concept of a near unitary matching of RGC to relay cell, these results are supported (at least in part) by previous studies that have suggested a modestly larger number of retinal inputs on relay cells than just one to two (Cleland and Lee 1985; Usrey et al., 1999; Sincich et al., 2007).

We are therefore left pondering why anatomical and functional studies produce such differing conclusions. Is this discrepancy a technical issue with the approaches applied? Certainly, this is a possibility, as all experimental approaches have shortcomings. Are these complex synapses leaky, so that the release of glutamate from one terminal activates all of the postsynaptic receptors within this synaptic cluster? Certainly, the later is a possibility since the presence and consequence of synaptic spillover has been demonstrated in both simulations and with experimental approaches at complex retinogeniculate synapses (Budisantoso et al., 2012). Do some classes of relay cells in mouse dLGN receive input from only one type of retinogeniculate synapse, so that some relay cells receive input from just one to two RGCs while other classes receive input from large numbers of RGCs? This possibility is supported in part by a heroic study by Sherman and colleagues in which a single retinal axon was labeled with HRP and its connectivity with four LGN relay cells was examined with serial electron microscopy (Hamos et al., 1987). The HRP-labeled axon accounted for 100% of the retinal inputs onto the proximal dendrites of one of the relay cells, but only 49%, 33%, and 2% of the retinal inputs of the other three relay cells, suggesting levels of convergence on relay cells may vary widely (Hamos et al., 1987). Moreover, the morphology of retinal terminals generated by this single HRP-labeled axon differed between the cells, suggesting a role for the postsynaptic neuron in determining the architecture of the retinogeniculate synapse (Hamos et al., 1987). However, our reconstructions identified cases in which single relay cells were innervated by both simple and complex retinogeniculate synapses, arguing against the possibility of different classes of relay cells receiving just one type of retinal synapse. Are “complex encapsulated” retinogeniculate synapses considerably weaker than the large, “simple encapsulated” synapses, so that their influence on postsynaptic activity is negligible? The reduced number of active zones and dendritic protrusions in retinal boutons associated with complex retinogeniculate, shown by us here and by Budisantoso et al. (2012) in the rat dLGN suggest that the strength of terminals may be weaker at complex synapses. Unfortunately, we do not know the answers to all of these questions yet, but one can certainly imagine a number of possibilities that will need to be addressed in future studies.

Results from our studies also raise interesting questions regarding how different classes of RGCs participate in retinal terminal clustering in dLGN. Only a single retinal axon was reconstructed that participated in both complex and simple type retinogeniculate synapses. While this may reflect a sampling issue, it may also indicate that different classes of RGCs generate different types of retinogeniculate synapses. Just as different classes of retinal axons arborize in unique domains of dLGN (Dhande and Huberman 2014; Hong and Chen 2011), it is tempting to speculate that some classes of retinal axons cluster their terminals into complex retinogeniculate synapses (like observed in Hong et al., 2014), while others do not use this mechanism of refinement and reorganization.

Finally, it is important to discuss an implication that these studies may have on using the retinogeniculate synapse as a model of activity-dependent refinement. Many groups use this model synapse to explore the cellular and molecular underpinnings of activity-dependent refinement at brain synapses (for examples, see Chen and Regehr, 2000; Hooks and Chen 2006; Hong et al., 2014; Schafer et al., 2012; Stevens et al., 2007). Such studies are based on the assumption that inputs from ~20 RGCs initially converge on relay cells, but that most of these inputs are eliminated during development. But, what if this is not the case? What if retinal convergence persists (at least anatomically) in the mature visual system? Our results lead us to think that this is the case and indicate we need to re-evaluate our understanding of the architecture and flow of visual information through retinogeniculate circuits.

## EXPERIMENTAL PROCEDURES

### Mice

Wild-type C57 mice were obtained from Charles River. *Calb2-cre* mice were obtained from Jackson Laboratory (stock #010774). All analyses conformed to NIH guidelines and protocols approved by the Virginia Polytechnic Institute and State University Institutional Animal Care and Use Committees.

### Intraocular Injections of Brainbow AAVs

The following brainbow AAVs were obtained from the University of Pennsylvania Vector Core (http://www.med.upenn.edu/gtp/vectorcore/): AAV9.hEF1a. lox.TagBFP.lox.eYFP.lox.WPRE.hGH-InvBYF (lot #V3809TI-R) and AAV9. hEF1a.lox.mCherry.lox.mTFP1.lox.WPRE.hGH-InvCheTF (lot #V3530TI-R). Each brainbow AAV is capable of driving the expression of two different fluorescent proteins (see Figure 1A). Intraocular injection of brainbow AAVs was performed as described previously for the intraocular delivery of cholera toxin subunit B (Jaubert-Miazza et al., 2005; Su et al., 2011). Briefly, mice were anesthetized with isoflurane vapors at P12-14. The sclera was pierced with a sharp-tipped glass pipette, and excess vitreous was drained. Another pipette, filled with a 1:1 mixture of both brainbow AAVs, was inserted into the hole made by the first pipette. The pipette containing the AAVs was attached to a Picospritzer and a prescribed volume (3–5 μl) of solution was injected into the eye. After 21 days, mice were euthanized and transcardially perfused with PBS and 4% paraformaldehyde, and retinas and brains were post-fixed in 4% paraformaldehyde for 12 hr. Fixed brains were coronally sectioned (80–100 μm) on a vibratome (Microm HM 650V, Thermo Scientific) and mounted in VectaShield (Vector Laboratories). Fixed retinas were either prepared as whole mounts or were sectioned on a Leica CM1850 cryostat (16-μm cross-sections) and in either case were mounted in VectaShield (Vector Laboratories) (Su et al., 2011). RGCs and retinal projections were analyzed from six animals. Images were acquired on a Zeiss LSM 700 confocal microscope and color analysis of maximum projections images was performed in Photoshop.

### Serial Block Face Scanning Electron Microscopy

Mice were transcardially perfused sequentially with PBS and 4% paraformaldehyde/2% glutaradehyde in 0.1 M cacodylate buffer. Brains were immediately removed and vibratomed (300-μm coronal sections) and dLGN were dissected. Tissues were then stained, embedded, sectioned, and imaged by Renovo Neural. Images were acquired at a resolution of 5 nm/pixel and image sets included >200 serial sections (with each section representing 75 nm in the z axis). SBFSEM data sets were 40 μm × 40 μm × 12–20 μm. Four data sets were analyzed (from a total of three P42 wild-type mice). Data sets were traced and analyzed in TrakEM2 (Cardona et al., 2012). Retinal terminals were identified (and distinguished from non-retinal terminals) by the presence of synaptic vesicles and pale mitochondria as previously described (Lund and Cunningham 1972; Bickford et al., 2010; Hammer et al., 2014). Synaptic sites were identified by the presence of active zones and postsynaptic densities. Analysis of data sets was performed independently by three researchers to ensure unbiased results.

## Supplementary Material

Supplemental material

## In Brief

Visual information is transmitted to the thalamus via retinal ganglion cell (RGC) axons. A near unitary matching of RGC to thalamic relay cell exists. Applying advanced imaging techniques, Hammer et al. challenge this fundamental concept by revealing a significantly larger number of retinal inputs on relay cells than previously appreciated.

## Highlights

- Brainbow-based technology can be used to differentially label retinal terminals
- Clusters of retinal terminals originate from numerous uniquely labeled RGCs
- Complex retinogeniculate synapses contain terminals from >10 distinct retinal axons
- A higher level of convergence exists on relay cells than previously appreciated
