# Supplementary material for: Multiple Retinal Axons Converge onto Relay Cells in the Adult Mouse Thalamus
Source: Cell Rep. Author manuscript; Available in PMC 2018 Jan 8. (PMC5757867; doi:10.1016/j.celrep.2015.08.003)
Supplement: Supplemental material [file NIHMS929594-supplement-Supplemental_material.pdf]

Multiple retinal axons converge onto relay cells in the adult mouse thalamus

Hammer, S., Monavarfeshani, A.,Lemon, T., Su, J. and Fox, M.A.

Supplemental Figures, Experimental Procedures and References

Supplemental Figure 1. Analysis of retinal bouton clustering in the “shell” and “core” of dLGN. A. Retinal terminals in the adult dLGN were immuno-labeled with antibodies directed against vesicular glutamate transporter 2 (VGluT2). High magnification images of terminals in the “shell” and “core” of dLGN are shown in A’ and A’’, respectively (and their exact positions are indicated in **A**). Arrows indicate clusters of VGluT2-positive retinal terminals, similar to those analyzed with brainbow AAVs in Figure 2. **B.** Retinal projections in the dLGN were labeled by intraocular injection of the anterograde tracer CTB. High magnification images of CTB-labeled terminals in the “shell” and “core” of dLGN are shown in B’ and B’’, respectively (and their exact positions are indicated in **B**). Arrows indicate clusters of CTB-positive retinal terminals, similar to those analyzed with brainbow AAVs in Figure 2. Scale bar in **B** = 200 μm from **A** and **B** and in **B’’** = 25 μm in **A’**,**A’’**, **B’** and **B’’**. **C-H**. SBFSEM images of 3 simple retinogeniculate synapses and 3 complex retinogeniculate synapses in the “core” region of dLGN. In **E** and **H**, the relay cell dendrites are pseudo-colored green, while retinal boutons contacting these dendrites are pseudo-colored red (in **E**) and blue, magenta, and yellow (in **H**). In **H**, the three boutons containing pale mitochondria and round synaptic vesicles originate from distinct arbors within the dataset traced. R – retinal terminal; D – relay cell dendrite. Scale bar in **H** = 2 μm for **C-H**.

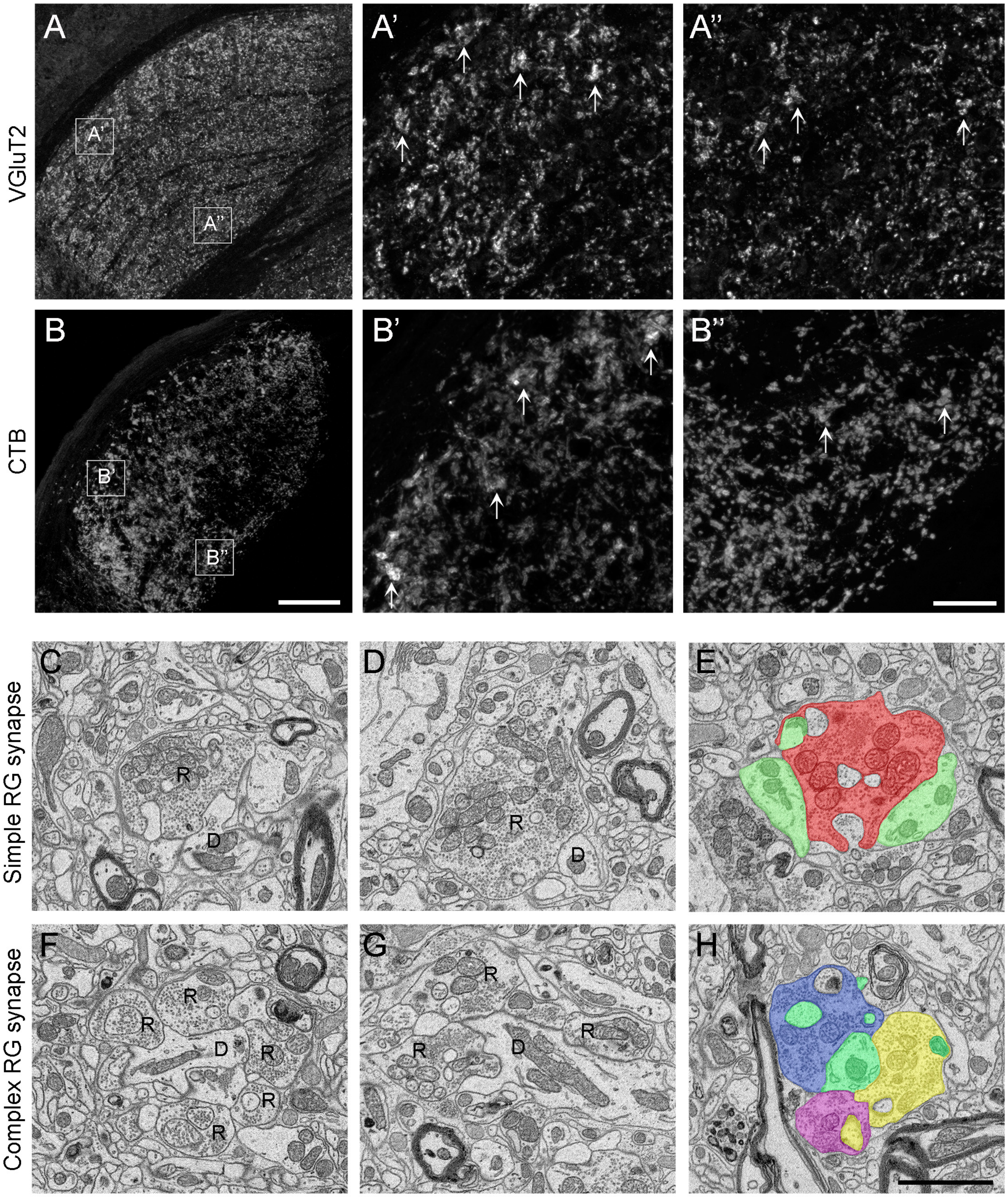
A
A'
A''
VGluT2
B
B'
B''
CTB
C
D
E
Simple RG synapse
F
G
H
Complex RG synapse
R
D

Supplemental Figure 2. Ultra-structural analysis of retinal axons contributing to simple and complex retinogeniculate synapses in dLGN. A. SBFSEM images of 4 simple retinogeniculate synapses and 4 complex retinogeniculate synapses in the “shell” region of dLGN. These panels provide non-pseudo-colored examples of retinal boutons similar to those pseudo-colored in Figures 3 and 4. Arrows highlight active zones with retinal terminals. Arrowheads highlight puncta adherens between retinal boutons and relay cell dendrites. R – retinal terminal; D – relay cell dendrite; p – dendritic protrusion. Scale bar = 2 μm **B-D**. Key features of simple and complex retinal synapses were quantified, including bouton diameter (at its widest point)(**B**), the number of dendritic protrusions into the retinal bouton (**C**), the number of active zones per bouton (**D**), and the active zone: bouton diameter ratio (**E**). Retinal boutons participating in simple retinogeniculate synapses had significantly larger diameters, more active zones, more dendritic protrusion and a higher active zone to bouton diameter ratio (which suggests that the increase in active zone number is not merely caused by larger bouton size).

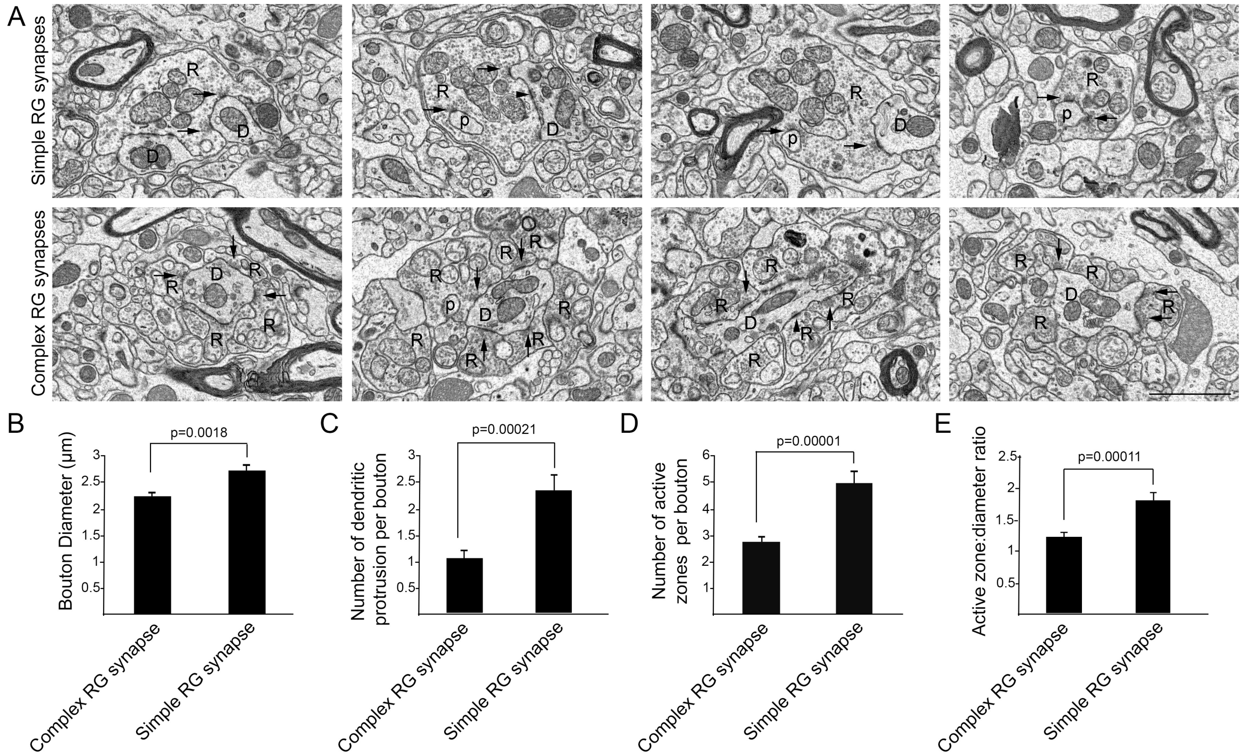
A
Simple RG synapses
Complex RG synapses
R
D
p
B
Bouton Diameter (µm)
p=0.0018
Complex RG synapse
Simple RG synapse
C
Number of dendritic protrusion per bouton
p=0.00021
D
Number of active zones per bouton
p=0.00001
E
Active zone:diameter ratio
p=0.00011

Supplemental Experimental Procedures

Mice

Wild-type C57 mice were obtained from Charles River. *Calb2-cre* mice were obtained from Jackson Laboratory (Stock #010774). All analyses conformed to National Institutes of Health guidelines and protocols approved by the Virginia Polytechnic Institute and State University Institutional Animal Care and Use Committees.

Intraocular injections of brainbow AAVs

The following brainbow AAVs were obtained from the University of Pennsylvania Vector Core (http://www.med.upenn.edu/gtp/vectorcore/):
AAV9.hEF1a.lox.TagBFP.lox.eYFP.lox.WPRE.hGH-InvBYF (lot #V3809TI-R) and AAV9.hEF1a.lox.mCherry.lox.mTFP1.lox.WPRE.hGH-InvCheTF (lot #V3530TI-R). Each brainbow AAV is capable of driving the expression of two different fluorescent proteins (see Figure 1A). Intraocular injection of brainbow AAVs was performed as described previously for the intraocular delivery of cholera toxin subunit B (Jaubert-Miazza et al., 2005; Su et al. 2011). Briefly, mice were anesthetized with isoflurane vapors at P12-14. The sclera was pierced with a sharp-tipped glass pipette and excess vitreous was drained. Another pipette, filled with a 1:1 mixture of both brainbow AAVs, was inserted into the hole made by the first pipette. The pipette containing the AAVs was attached to a Picospritzer and a prescribed volume (3–5 µl) of solution was injected into the eye. After 21 days, mice were euthanized, transcardially perfused with PBS and 4% paraformaldehyde, and retinas and brains were post-fixed in 4% paraformaldehyde for 12 hours. Fixed brains were coronally sectioned (80-100 μm) on a vibratome (Microm HM 650V, Thermo Scientific) and mounted in ProLong Gold (Invitrogen).

Fixed retinas were either prepared as whole-mounts or were sectioned on a Leica CM1850 cryostat (16μm cross-sections) and in either case were mounted in ProLong Gold (Invitrogen)(Su et al. 2011). RGCs and retinal projections were analyzed from 6 animals. Images were acquired on a Zeiss LSM 700 confocal microscope and color analysis of maximum projections images was performed in Photoshop.

Serial Block Face Scanning Electron Microscopy

Mice were transcardially perfused sequentially with PBS and 4% paraformaldehyde / 2% glutaradehyde in 0.1M cacodylate buffer. Brains were immediately removed, vibratomed (300 μm coronal sections) and dLGN were dissected. Tissues were then stained, embedded, sectioned and imaged by Renovo Neural Inc. (Cleveland, OH). Images were acquired at a resolution of 5 nm/pixel and image sets included > 200 serial sections (with each section representing 75 nm in the z axis). SBFSEM data sets were 40μm x 40 μm x 12-20 μm. 4 data sets were analyzed for each region (from a total of 3 P42 wild-type mice). Data sets were traced and analyzed in TrakEM2 (Cardona et al. 2012). Retinal terminals were identified (and distinguished from non-retinal terminals) by the presence of synaptic vesicles and pale mitochondria as previously described (Lund and Cunningham 1972; Bickford et al. 2010; Hammer et al. 2014). Synaptic sites were identified by the presence of active zones and postsynaptic densities. Analysis of data sets was performed independently by three researchers to ensure unbiased results.

Anterograde labeling of retinal axons with cholera toxin subunit B (CTB):

Intraocular injection of cholera toxin subunit B (CTB) was performed as described previously (Su et al. 2011; Hammer et al. 2014). CTB was delivered into only the right eyes of P25 mice in these studies. 2 days after injection, mice were perfused with PBS and 4% paraformaldehyde (in PBS) and brains were post-fixed in 4% paraformaldehyde (in PBS) overnight at 4°C. Fixed brains were washed in PBS, then immersed in 30% sucrose (in PBS) for 2-3 days. 14-16 μm coronal sections were obtained on a Leica CM1850 cryostat and thaw-mounted onto SuperFrostPlus slides. Sections were covered with VectaShield (Vector Laboratories, Burlingame, CA) and cover-slipped. Images of dLGN were acquired on a Zeiss LSM 700 confocal microscope.

Immunohistochemistry:

Immunohistochemistry (IHC) was performed as previously described (Su et al. 2011; Hammer et al. 2014). Briefly P25 mice were perfused with PBS and 4% paraformaldehyde (in PBS) and brains were post-fixed in 4% paraformaldehyde (in PBS) overnight at 4°C. Fixed brains were washed in PBS, then immersed in 30% sucrose (in PBS) for 2-3 days. 14-16 μm coronal sections were obtained on a Leica CM1850 cryostat and thaw-mounted onto SuperFrostPlus slides. Sections were incubated in blocking buffer (2.5% normal goat serum, 2.5% bovine serum albumin, 0.1% Triton X-100 in PBS) for 30 minutes and then incubated in primary antibody (rabbit anti-VGluT2, 1:500, Synaptic Systems, Inc.) in blocking buffer, over night at 4°C. Aftering washing in PBS, fluorescently-conjugated secondary antibodies (Invitrogen) were applied to sections for 1 hour at room temperature followed by mounting in VectaShield and imaging on a Zeiss LSM 700 confocal microscope.

Supplemental References:

Bickford, M. E., Slusarczyk, A., Dilger, E. K., Krahe, T. E., Kucuk, C., and Guido, W. (2010). Synaptic development of the mouse dorsal lateral geniculate nucleus. J Comp Neurol 518, 622-635.

Cardona, A., Saalfeld, S., Schindelin, J., Arganda-Carreras, I., Preibisch, S., Longair, M., Tomancak, P., Hartenstein, V., and Douglas, R. J. (2012). TrakEM2 software for neural circuit reconstruction. PLoS One 7, e38011.

Hammer, S., Carrillo, G. L., Govindaiah, G., Monavarfeshani, A., Bircher, J. S., Su, J., Guido, W., and Fox, M. A. (2014). Nuclei-specific differences in nerve terminal distribution, morphology, and development in mouse visual thalamus. Neural Dev 9, 16.

Jaubert-Miazza, L., Green, E., Lo, F. S., Bui, K., Mills, J., and Guido, W. (2005). Structural and functional composition of the developing retinogeniculate pathway in the mouse. Vis Neurosci 22, 661-676.

Lund, R. D., and Cunningham, T. J. (1972). Aspects of synaptic and laminar organization of the mammalian lateral geniculate body. Invest Ophthalmol 11, 291-302.

Su, J., Haner, C. V., Imbery, T. E., Brooks, J. M., Morhardt, D. R., Gorse, K., Guido, W., and Fox, M. A. (2011). Reelin is required for class-specific retinogeniculate targeting. J Neurosci 31, 575-586.
